# Elucidating the role of stacking faults in TlGaSe_2_ on its thermoelectric properties

**DOI:** 10.1038/s41699-025-00569-x

**Published:** 2025-06-06

**Authors:** Tigran Simonian, Ahin Roy, Akash Bajaj, Rui Dong, Zheng Lei, Zdeněk Sofer, Stefano Sanvito, Valeria Nicolosi

**Affiliations:** 1 School of Chemistry, Trinity College Dublin, Dublin, Ireland; 2 Centre for Research on Adaptive Nanostructures and Nanodevices (CRANN), Trinity College Dublin, Dublin, Ireland; 3 Materials Science Centre, Indian Institute of Technology Kharagpur, Kharagpur, West Bengal India; 4 School of Physics, Trinity College Dublin, Dublin, Ireland; 5 Department of Inorganic Chemistry, University of Chemistry and Technology Prague, Prague, Czech Republic

**Keywords:** Thermoelectrics, Transmission electron microscopy, Electronic structure, Two-dimensional materials, Structural properties

## Abstract

Thermoelectric materials are of great interest for heat energy harvesting applications. One such promising material is TlGaSe_2_, a 2D-layered, *p*-type semiconducting ternary chalcogenide. Recent reports show it can be processed as a thin film, opening the door for large-scale commercialization. However, TlGaSe_2_ is prone to stacking faults along the [001] stacking direction and their role in its thermoelectric properties has not yet been understood. Herein, TlGaSe_2_ is investigated via (scanning) transmission electron microscopy and first-principles calculations. Stacking faults are found to be present throughout the material, as density functional theory calculations reveal a low stacking fault energy of ~12 mJ m^−2^. Electron transport calculations show an enhancement of thermoelectric power factors when stacking faults are present. This implies the presence of stacking faults is key to the material’s excellent thermoelectric properties along the [001] stacking direction, which can be further enhanced by doping the material to carrier concentrations of ~10^19 ^cm^−3^.

## Introduction

Thermal management is a prescient issue in electronic devices such as the high-performance CPUs and GPUs, which are the foundation of current advances in machine learning^1–3^, computational chemistry^4–7^, climate modeling^8–11^, etc., as inefficient heat transfer can lead to overheating and damage to the device. This is especially important as the transistors in such devices approach the nanometer and atomic scales^12^. A mitigation procedure for overheating is “thermal throttling”, where the clock speed of the device is reduced to protect it from potential damage due to overheating at the cost of performance^13–16^. To avoid this and retain performance, the governance of this excess heat is often managed through a combination of heat sinks, fans, or liquid-cooling systems, which require additional electrical energy leading to increased operating costs. Conversion of this excess heat back into electrical energy would provide a means of powering the cooling systems for the devices, reducing overall costs and increasing efficiency.

Thermoelectric materials could be used to achieve this goal. These materials have low thermal conductivity yet possess high electrical conductivity, allowing them to convert heat energy into electrical energy when there is a thermal gradient across the material^17–20^. The performance of thermoelectric materials is compared via the dimensionless figure of merit *ZT*, which is defined as:

$${ZT}=\,\frac{{S}^{2}\sigma }{\kappa }T,$$

where *S* is the Seebeck coefficient (V K^−1^), σ is the electrical conductivity [(Ω m)^−1^], κ is the thermal conductivity [W (m K)^−1^], and *T* is the temperature (K)^21^. For comparison, bulk Si has a *ZT* of approximately 0.01 at 300 K due to its high thermal and electrical conductivity^22^. Many commercially available thermoelectric devices are made from PbTe- and Bi_2_Te_3_-based alloys, with *ZT* ∼ 0.7–0.8 at 300 K^23–27^, but these devices have low conversion efficiencies of only 8% at ambient temperatures^24,28^. The family of Bi_x_Te_y_ nanowires has some of the highest reported *ZT* values at room temperature, where *ZT* ∼ 0.8–1.5, but manufacturing devices with these materials at scale has been a challenge until recently^29,30^.

Other materials such as clathrates^31^ and skutterudites^32^ follow the concept of “phonon glass, electron crystal” in order to achieve low thermal conductivity while maintaining electrical performance^25,26,33^. These materials contain weakly bound atoms in the cavities of the unit cell, enabling anharmonic “rattling” that, in turn, leads to the phonon softening^34–37^. While these materials possess *ZT*> 1.3, these typically have high operating temperatures of at least 600 K^25,26,33^ making them unsuitable for many consumer-grade applications^25^.

Another potential material for high-*ZT*, ambient temperature thermoelectric devices is the layered 2D ternary chalcogenide TlGaSe_2_. Unlike most layered thermoelectric materials which have poor thermoelectric properties at ambient temperature^38–40^, TlGaSe_2_ has a high *ZT* of approximately 0.8 at 300 K^41–43^. TlGaSe_2_ is also a *p*-type semiconductor with a band gap of ∼ 1.95–2.2 eV^42–47^, far larger than that of the commercial PbTe- or Bi_2_Te_3_-based alloy thermoelectric devices^48,49^, allowing for optoelectronic applications such as X-ray and gamma-ray photodetectors^45,50^.

Recently, thin films of TlGaSe_2_ have been reported^50^, allowing for the production of thermoelectric devices at large scales. The layered nature of the material leads to highly anisotropic thermoelectric properties^42,51^, which may be exploited for controlled thermal transfer through a device^52,53^. This layering also means that the material is susceptible to planar defects such as stacking faults, as it had been noted in previous reports^54,55^. However, it has yet to be established if these stacking faults are intrinsic to the system or are a by-product of external stimuli such as heat.

Stacking faults in other layered materials have been shown to dampen the thermal conductivity along the stacking direction, which can increase the *ZT* of the material^56–59^. While the presence of Se vacancies is theorized to greatly hamper the thermoelectric properties of TlGaSe_2_, the role of stacking faults on this property has yet to be established to date^41^. In this work, TlGaSe_2_ was characterized via high-angle annular dark field scanning transmission electron microscopy (HAADF-STEM) imaging and selected area electron diffraction (SAED) patterns, which were then correlated to simulations. The nature of the stacking faults in TlGaSe_2_ was investigated using density functional theory (DFT) calculations of the stacking fault energies. The role of these faults on the thermoelectric properties was furthermore probed via electron transport calculations, highlighting an enhancement due to the presence of the stacking faults and when the carrier concentration is increased.

## Results

### Characterization of stacking faults in TlGaSe_2_

**Fig. 1:**
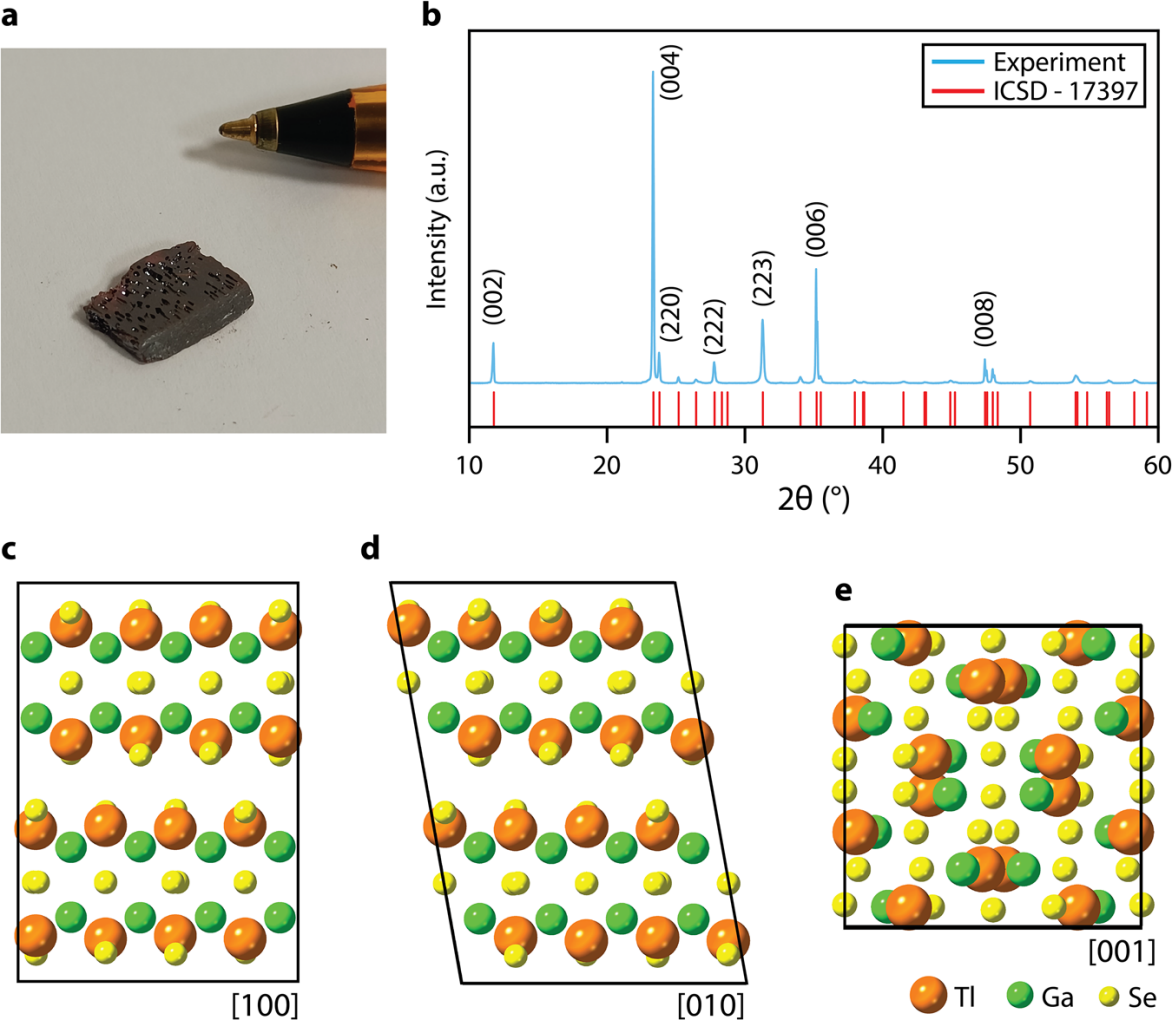
Crystal structure of TlGaSe_2_. **a** Photograph of bulk TlGaSe_2_, with tip of ballpoint pen for scale; **b** XRD pattern of TlGaSe_2_ (blue), along with appropriate fitting of the structure (red) from ICSD – 17397; **c**–**e** Monoclinic unit cell of TlGaSe_2_, shown along [100], [010] and [001] zone axes, respectively.

Bulk crystals of TlGaSe_2_ were grown via vacuum melt growth and its monoclinic structure (space group *C12/c1*; ICSD – 17397)^60^ was confirmed via X-ray diffraction (XRD) (Fig. 1a, b; see Methods for details). The lattice parameters were determined to be *a* = *b* = 10.772 Å, c = 15.636 Å, β = 100.06°.

The unit cell consists of Ga_4_Se_10_ units, each containing four corner-sharing GaSe_2_ tetrahedra (Fig. 1c–e). These form two layers within each unit cell which are aligned parallel to the (001) plane, and perpendicular to the [114] direction. The layers are connected to each other through the Tl^+^ ions located in the center of the Se_6_ trigonal cavities between the layers^54,55,61,62^.

**Fig. 2:**
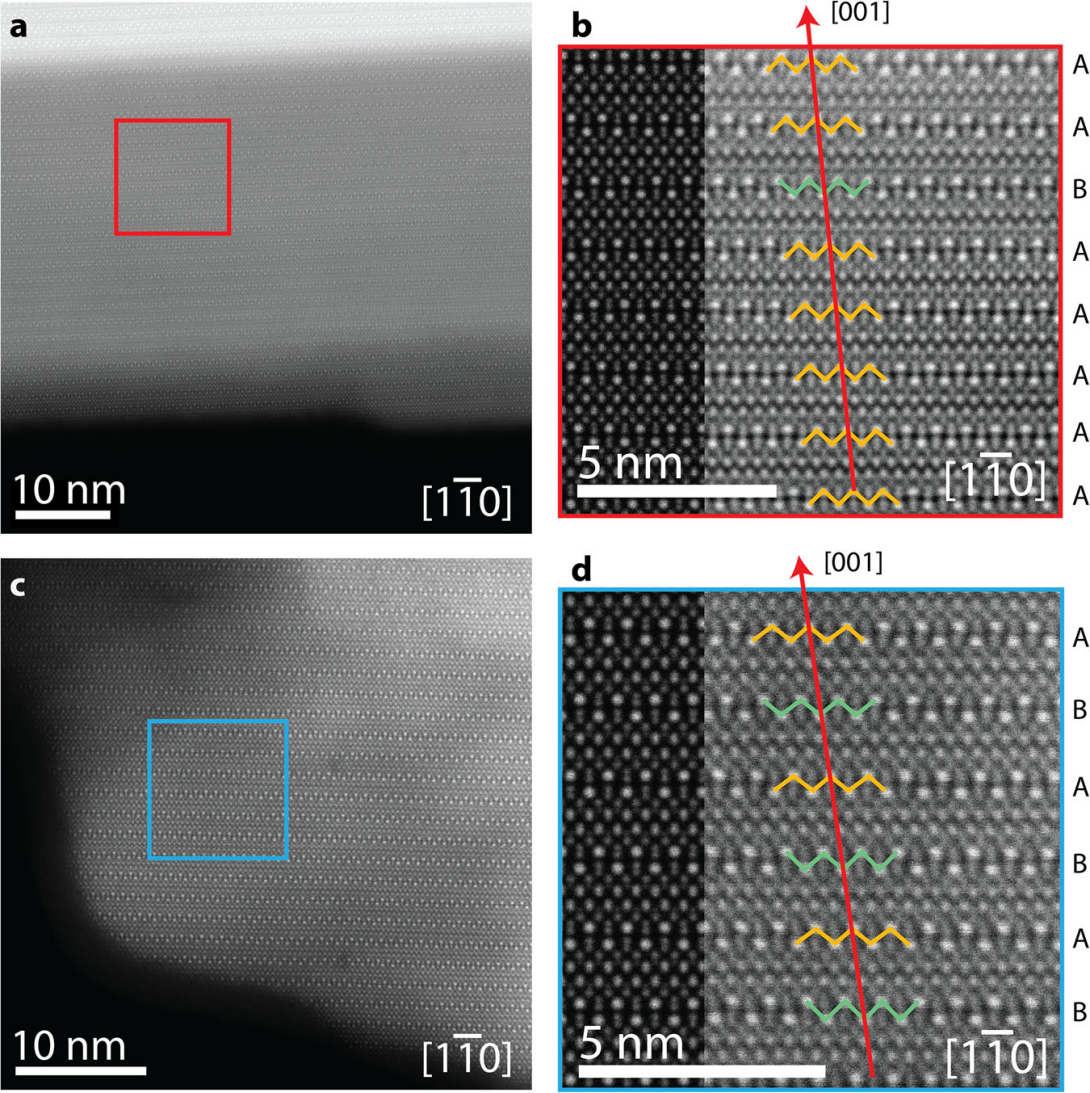
STEM imaging of TlGaSe_2_. HAADF-STEM images (**a**, **c**) of TlGaSe_2_ samples along the [1$\bar{1}$0] zone axis, with magnified regions shown in (**b**) and (**d**), respectively. Red arrows are along the [001] direction, highlighting the stacking faults along the stacking direction. The stacking order in (**b**) and (**d**) is shown along the right-hand side with green zig-zag lines to highlight the stacking faults, while multislice simulations of each image are shown along the left-hand side.

HAADF-STEM imaging of TlGaSe_2_ samples along the [1$\bar{1}$0] zone axis (Fig. 2) highlights the layered nature of the material. Following along the stacking direction of [001] reveals many stacking faults throughout the sample (Fig. 2c, d). Multislice simulation of the HAADF-STEM images (Fig. 2b, d) confirms the presence of stacking faults. Though some short-range ordering of the layers was present, no long-range ordering was observed in any of the samples of TlGaSe_2_ studied, which is in line with previous literature^54,55^.

**Fig. 3:**
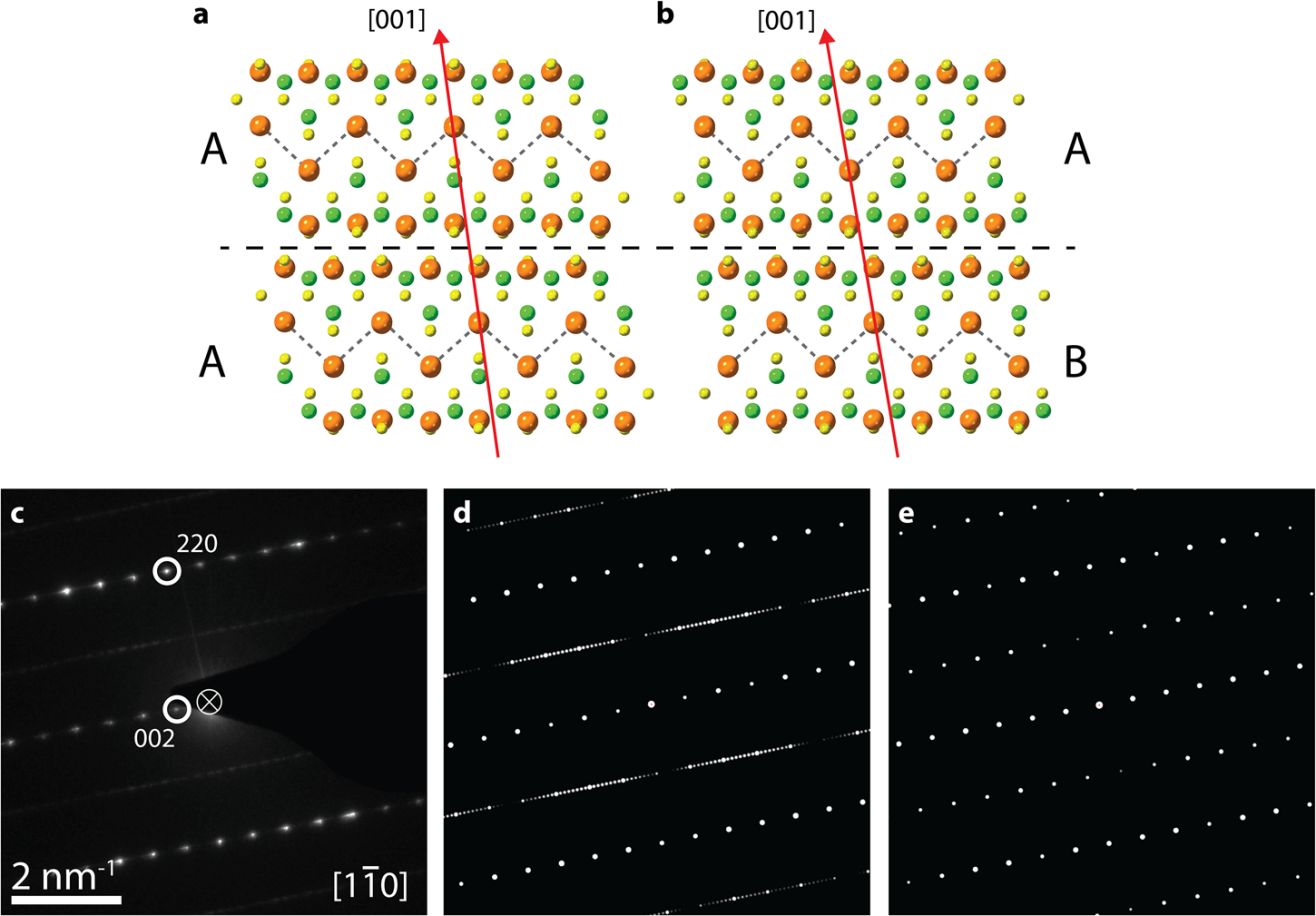
SAED studies of TlGaSe_2_ stacking faults. *AA-* (**a**) and *AB-* (**b**) stacking of TlGaSe_2_ seen along [1$\bar{1}$0] zone axis. Red arrows showing the [001] direction and polyhedra around GaSe_2_ units are shown to highlight the stacking order. **c** Selected area electron diffraction (SAED) pattern of TlGaSe_2_ along [1$\bar{1}$0] zone axis. Dynamical diffraction simulations, along the same zone axis and same scale as (**c**), of the SAED pattern (**d**) and of a structure in bulk, *AA*-stacking only (**e**). The circle with a cross in (**c**) indicates the central beam, which has been covered by the beam blocker.

The stacking order in TlGaSe_2_ exists between unit cells along the [001] direction and appears in one of two forms: *AA* and *AB* (Fig. 3a, b). The *AA*-stacking is considered the bulk stacking mode and can be described as a pseudotranslation of a unit cell with respect to the cell below it along the [110] direction:

$${s}_{1}=\frac{1}{4}{\boldsymbol{a}}+\frac{1}{4}{\boldsymbol{b}},$$

where ***a*** and ***b*** are the lattice vectors. Stacking faults in TlGaSe_2_ are classified as *AB*-stacking (Fig. 3b) when there is a 90° rotation of a unit cell with respect to the cell below it about the [1$\bar{1}$4] direction. Conveniently, this too can be considered a pseudotranslation along the [110] direction:

$${s}_{2}=\frac{1}{4}{\boldsymbol{a}}-\frac{1}{4}{\boldsymbol{b}}$$

To corroborate the short-range order seen in the HAADF-STEM imaging, SAED was performed on the same area of the sample shown in Fig. 2a. Diffuse “streaking” was observed along the (*hhl*) directions where *h* = odd (Fig. 3c)^54^. The presence of “streaks” in these diffraction patterns can indicate the presence of stacking faults in the structure^63^. As the number of stacking faults increases, the number of additional spots should increase as the diffuse intensities are split symmetrically around the positions of the Bragg intensities with *h*$\bar{h}$*l*. However, low numbers of stacking faults imply extended local domains, which can lead to the smearing or “streaking” of the spots along the c* direction^55,64^.

The lack of fully continuous streaks in Fig. 3c implies some short-range order to the stacking of the layers in this part of the sample^54^. To confirm this, a TlGaSe_2_ model consisting of the stacking order in Fig. 2a was used to simulate a SAED pattern using dynamical diffraction simulations (Fig. 3d; see Methods for details)^54,55,64^. For comparison, a TlGaSe_2_ model with only *AA*-stacking, i.e. bulk stacking mode, was also simulated under the same conditions (Fig. 3e). The simulated SAED in Fig. 3d aligns closely with the experimental SAED, while the simulation in Fig. 3e contains no “streaks” at all. However, some discrepancies in the streaking in Fig. 3d do exist. This is because the smallest SAED aperture available on the TEM used was 10 µm in diameter, an area at least 10x larger than that seen in the HAADF-STEM image in Fig. 2a. The area of interest was along the edge of the sample, and the sample position and aperture were aligned such that the field of view only contained this edge. However, ensuring this was difficult and hence, parts of the sample beyond the area of interest would have been exposed to the electron beam, contributing their stacking order to the diffraction pattern.

Previous XRD studies have shown that these short-range domains can be on average four unit cells thick^54^. However, the limited statistics of TEM means making the same conclusions is difficult. Nonetheless, the streaking does confirm the short-range nature of the stacking order of TlGaSe_2_ as seen in Fig. 2 and as had been previously noted^54,55^.

The ubiquity of these stacking faults required further examination as it was not clear whether they are intrinsic to the system (i.e. appear at the growth stage) or must be extrinsically induced via external stimuli such as heating, etc. To investigate this aspect, the stacking fault energy was calculated via DFT using the following equation:

$${\gamma }_{{SFE}}=\frac{{E}_{{fault}}-{E}_{{bulk}}}{2A}$$

where *E*_*fault*_ is the ground state energy of the faulted system, *E*_*bulk*_ is the ground state energy of the bulk system, and *A* is the area of the stacking fault^65,66^. Here, the area of the stacking fault is the area of the (001) plane of a unit cell. The ground state energy per unit cell of the bulk stacking order (*AA*) and a fully faulted (*AB*) system were calculated (see Methods for details), with the resulting stacking fault energies evaluated using the equation above. To establish whether an equilibrium stacking fault distance may exist, an *AAB*-ordered structure was also trailed. These relaxed structures are shown as CIF structure files in Supplementary Dataset 1.

After calculating the ground state energies per unit cell (Supplementary Table 1), the stacking fault energies are calculated to be 12.70 mJ m^−2^ when comparing the bulk stacking to the *AB*-stacked structure. This is remarkable as such low stacking fault energies are typically only seen in medium- and high-entropy alloys^67–72^. This would imply that the stacking faults in TlGaSe_2_ may have occurred during growth^73^, however, preparing the sample for TEM characterization may have also caused some of these faults to occur (see Methods for details). Nonetheless, the low value of the stacking fault energy and the minimal change in the value to 12.67 mJ m^−2^ when an *AAB*-stacking order was used instead, can help to explain why long-range ordering was not observed in the experimental data, or in previous studies^54,55^. The low values of the stacking fault energies may be due to the nature in which the stacking faults in TlGaSe_2_ occur. Unlike other 2D materials which have many polytypes such as GaSe^74^ or MoS_2_^75–77^, the stacking of the layers in TlGaSe_2_ can only exist in two states, *AA* or *AB*^55^ (Fig. 3a, b). Therefore, the *AB*-stacking fault can also be treated as twinning, and materials which have a high abundance of twinning typically also have low stacking fault energies^69,78^.

### First-principles calculations of thermoelectric properties

The high concentration of stacking faults in the materials may therefore play a role in its impressive thermoelectric properties. Similar ternary chalcogenide materials such as TlInSe_2_^79^, Cu_2_TiTe_3_^80^, and TlAgSe^81^ were experimentally characterized and were shown to have impressive thermoelectric properties. Accordingly, stacking faults in similar thermoelectric materials such as InGe_2_Te_6_^82^ have been shown, both theoretically and experimentally, to reduce thermal conductivity and thus enhance thermoelectric properties^56–59^. However, for TlGaSe_2_, the abundance of stacking faults makes it also very cumbersome to isolate large enough sections of fault-free material to conduct electrical and thermal conductivity measurements. This is highlighted by the fact that, to the best of our knowledge, there has only been one experimental study on the thermoelectric properties of TlGaSe_2_, via the measurement of its Seebeck coefficient^42^. However, this study did not consider the presence of stacking faults in the material, nor is it clear which crystallographic direction the measurements were taken from. Trying to obtain a priori knowledge of which sections of the material are fault-free is also non-trivial. Hence density functional theory calculations were further used to qualitatively probe the role of the stacking faults on the thermoelectric properties of TlGaSe_2_.

The numerator of the thermoelectric figure of merit *ZT* can be used as a measure of thermoelectric power, which is useful as thermal transport measurements and calculations are non-trivial regardless of the material system^83–87^. To investigate this aspect, the electronic conductance for a bulk (*AA*-stacking) structure and one with a stacking fault were first calculated using first-principles ballistic transport calculations via DFT + NEGF (Supplementary Fig. S1; see Methods for details). There appears to be a negligible difference in the ballistic conductance along the [001] direction when a stacking fault is introduced. However, with Fermi level downshifts of at least ~0.5 eV below the neutrality point, the stacking fault does appear to suppress the conductance.

**Fig. 4:**
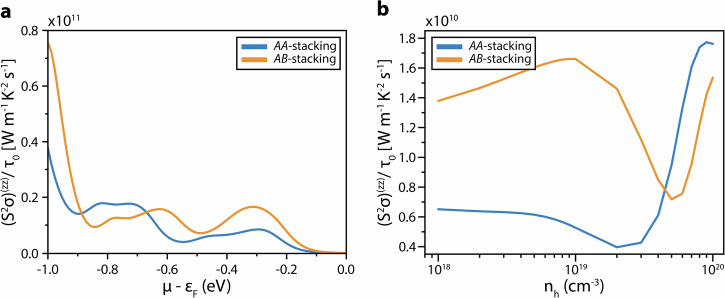
Thermoelectric power factor calculations of TlGaSe_2_ along the [001] stacking direction at 300 K. Thermoelectric power factors (**a**) of bulk (*AA*) (blue) and *AB*-stacking (orange) of TlGaSe_2_ along the [001] stacking direction. The power factors as a function of hole concentration in the system is shown in (**b**).

In order to gain a full understanding of the electronic properties relevant to thermoelectricity, the thermoelectric power factor, *S*^*2*^*σ*, was calculated for both *AA-* and *AB*-type stacking of TlGaSe_2_ within the semiclassical Boltzmann transport formalism under the constant relaxation time (τ_0_) approximation^88^ (Fig. 4a; see Methods for details). Under this approximation, only the Seebeck coefficient is independent of τ_0_. Thus, the power factor is reported using the scaled metric, *S*^*2*^*σ/τ*_*0*_, at 300 K.

For Fermi level downshifts of less than 0.2 eV below the neutrality point, the magnitude of *S*^*2*^*σ/τ*_*0*_, as well as the difference between the two stacking systems along the [001] stacking direction (i.e. *zz*-components), is small. This would be the case for intrinsic TlGaSe_2_ where the reported acceptor concentrations are only as high as 10^15 ^cm^−3^ ^41,42^. However, with further Fermi level downshifts, especially around ~0.6 eV below the neutrality point, the *AB*-configuration exhibits a much larger *S*^*2*^*σ/τ*_*0*_. This is especially possible if acceptor carrier concentrations of the order of 10^19 ^cm^-3^ can be achieved (Fig. 4b). At this concentration, the Seebeck coefficient along the [001] stacking direction more than doubles when in the presence of stacking faults, from 42 µV K^−1^ to 110 µV K^−1^. While this value is not as high as some of the commercially available thermoelectric materials, such as Bi_2_Te_3_, which has a Seebeck coefficient of 260 µV K^−1^ at 300 K^46,89^, it is in line with other 2D-layered tertiary chalcogenides such as Cu_2_TiTe_3_, with a Seebeck coefficient of 100 µV K^−1^ at 300 K^80^. As such, presence of stacking faults may be contributing to the latter’s impressive thermoelectric response as well.

The *σ/τ*_*0*_ of along the [001] stacking direction of TlGaSe_2_ halves when in *AB*-stacking, from 0.31 ×10^19^ (Ω m)^−1^ s^−1^ to 0.14 ×1019 (Ω m)^−1^ s^−1^. However, this leads to a threefold increase in the thermoelectric power factor (*S*^*2*^*σ/τ*_*0*_) for *AB*-stacking, from 0.53 ×1010 W m^−1^ K^−2^ s^−1^ to 1.66 ×1010 W m^−1^ K^−2^ s^−1^. Given the constant relaxation time approximation used in these calculations, it is difficult to compare these power factors with other materials, nonetheless, it shows a significant increase in thermoelectric properties when stacking faults are accounted for.

The effect of the *p*-type doping level on these power factors in the presence of stacking faults is also quite dramatic along the directions transverse to the stacking fault (Supplementary Figs. S2–S5). These calculations also show a clear anisotropy of the thermoelectric properties, where the *AB*-stacking enhances the thermoelectric power factors along the *zz*- and *xx-*directions, while it is suppressed along the *yy-*direction.

**Fig. 5:**
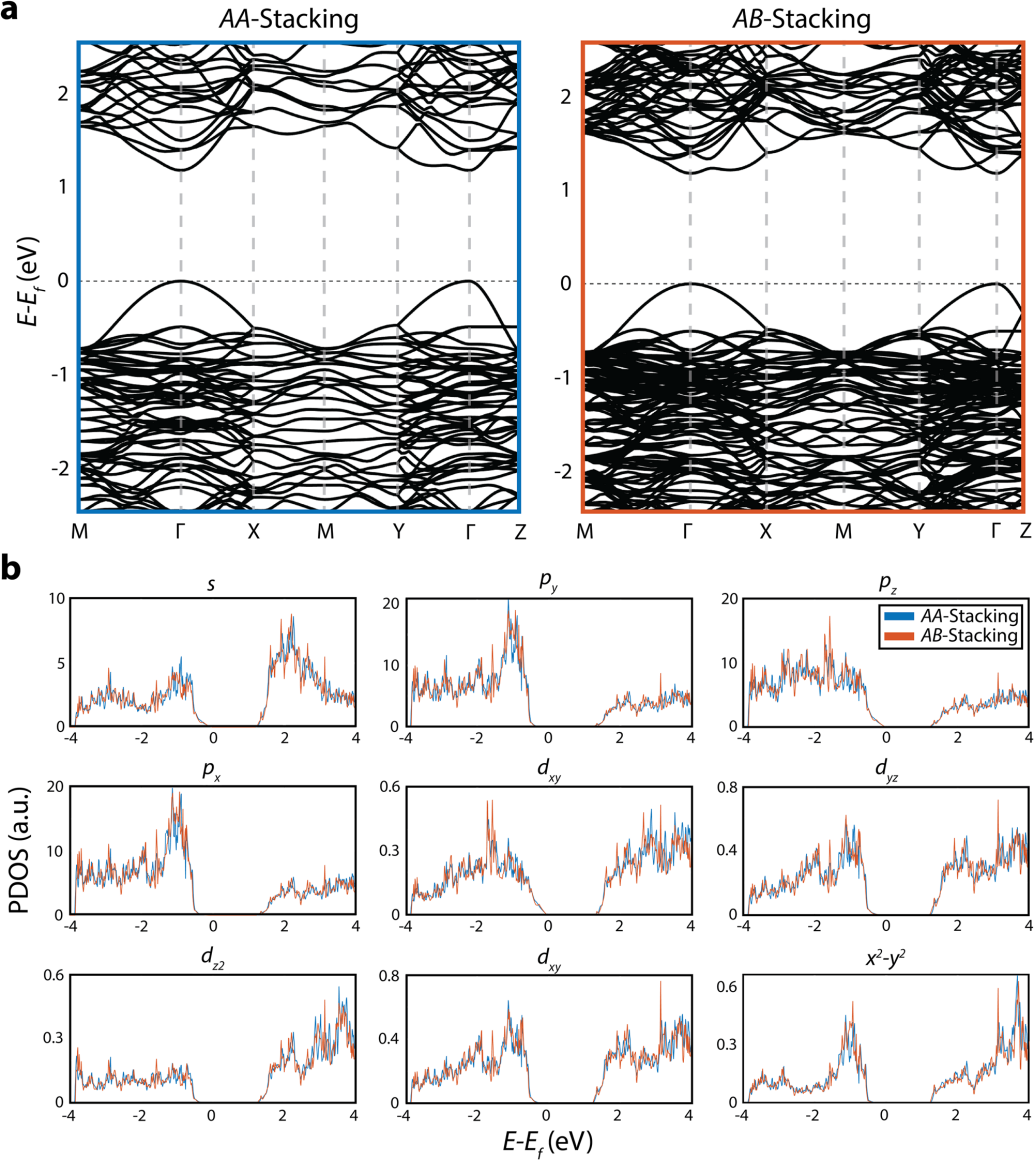
Electronic band structures and projected density of states (PDOS) of *AA*- and *AB*-stacked TlGaSe_2_. Electronic band structures of bulk (*AA*) (blue) and *AB*-stacking (orange) of TlGaSe_2_ are shown in (**a**). The projected density of states for both stacking types are shown in (**b**), presented here by each orbital type present in TlGaSe_2_.

To understand the origin of the difference in transport properties between *AA*- and *AB*-stacking, the band structure and projected density of states (PDOS) of both were calculated (Fig. 5; see Methods for details). From the band structures (Fig. 5a), the effective mass of the charge carriers can be derived from the bands extrema using the finite difference method^90,91^. As TlGaSe_2_ is an intrinsic *p*-type semiconductor^92^, the hole effective mass along the [001] stacking direction was found from the valence band maxima to be 0.11 *m*_0_ for *AA*-stacking and 0.10 *m*_0_ for *AB*-stacking, where *m*_0_ is the mass of a free electron. Similar results are seen for the in-plane directions, and for the electron effective masses (Supplementary Table 2). Such low effective masses are further indications of the noteworthy thermoelectric properties of TlGaSe_2_^93^, however the negligible change does not help to explain the superior thermoelectric performance of *AB*-stacking over *AA*-stacking.

A similar observation can be obtained from the PDOS (Fig. 5b); other layered thermoelectric materials such as Ca_3_Co_4_O_9_ show an increase in out-of-plane Co-O *p*_*z*_ states when a stacking fault is present, which can be observed experimentally through the use of electron energy loss spectroscopy^94^. However, for TlGaSe_2_, there are no significant shifts or increases in the PDOS for *AB*-stacking when compared to bulk *AA*-stacking. Nevertheless, the minor changes present in the band structure when a stacking fault is introduced can lead to varying transport properties, especially at elevated temperatures and carrier concentrations. A similar phenomenon has been seen in other 2D-layered ternary chalcogenide materials such as Cr_2_Ge_2_Te_6_^95^. Additional research would need to be performed, such as more accurate band structure calculations using hybrid functionals^96^ and thermal transport calculations^57,58,97^, to fully understand the role of stacking faults on the thermoelectric properties of TlGaSe_2_, however such work is outside the scope of the current studies.

## Discussion

In summary, the stacking faults in TlGaSe_2_ have been found to possess a very low stacking fault energy which form short-range domains throughout the structure. This was evident from the HAADF-STEM imaging and SAED studies, which were correlated to multislice simulation and dynamical diffraction calculations, respectively. While more work is required to concretely determine the origin of the difference in transport properties between *AA*-stacked and *AB*-stacked TlGaSe_2_, it is clear that stacking faults appreciably enhance the material’s thermoelectric properties, which are then further enhanced if one were to increase the *p*-type carrier concentration via doping. It is envisioned that this research would lead to further pursuits of using TlGaSe_2_ in a variety of thermoelectric applications.

## Methods

### Sample preparation

High purity elements (Tl (99.99%, granules, Changsha Asian Light Economic Trade Co., China), Ga (99.9999%, granules, Wuhan Xinrong New Materials Co., China) and Se (99.9999%, 2–4 mm granules, Wuhan Xinrong New Materials Co., China)) were used to synthesize bulk TlGaSe_2_. Stoichiometric amounts of the elements, corresponding to 20 g of TlGaSe_2_, was placed into a quartz ampoule (25 mm × 120 mm, 3 mm wall thickness) and melt sealed under vacuum (∼10^−3 ^Pa) using a diffusion pump. The ampoule was then heated to 850 °C using a heating rate of 1 °C min^−1^, held at that temperature for 5 h, and then cooled to room temperature at a rate of 0.2 °C min^-1^.

### X-ray diffraction

X-ray diffraction was performed using a Bruker D8 Discover diffractometer on a silicon holder with a Cu Kα source. The measured range was 5–90 °2θ, with a step size was set to 0.012 °2θ and at a rate of 0.2 s/step.

### (Scanning) transmission electron microscopy

Single crystals of the material were placed between several sheets of filter paper and ground up with a mortar and pestle into a fine powder, then dispersing in isopropyl alcohol. Samples were drop-cast onto copper, 400-grid lacey carbon TEM grids (Ted Pella, Inc.), with subsequent baking in a vacuum oven at approx. 80 °C for 24 h. HAADF-STEM imaging and SAED patterns were performed on a non-aberration corrected FEI Titan operating at 300 kV. HAADF-STEM imaging was performed with a convergence angle of 10 mrad and a collection angle range of 40–200 mrad. The probe current was approx. 20 pA and imaging was performed with a 10 µs dwell time with 1024 × 1024 pixels.

### Multislice and diffraction simulations

For all simulations in this work, the stacking faults were constructed with the ATOMMAN python package^98^, using the stacking fault pseudotranslation equations in the main paper. The ReciPro crystallography software was used to simulate the diffraction patterns^99^. Multislice simulations of HAADF-STEM images was performed via the abTEM code^100,101^, with roughly similar parameters as the imaging conditions of the reference images to provide a qualitative understanding of the structure.

### Stacking fault energy calculation

Calculation of the stacking fault energy was performed using density functional theory via the VASP code with projector augmented wave pseudopotentials^102,103^. Atomic structures were optimized using the PBE-GGA functional^104^ with a Hellmann–Feynman force criterion of 10^−2 ^eV/Å and a 520 eV cutoff energy. Calculations of the stacking fault energy were then performed with a cutoff energy of 400 eV and a 10^−5 ^eV convergence criteria. A 6 × 6 × 4 k-point grid was used in all calculations.

### Electric transport calculation

DFT-level quantum transport calculations were carried out on the relaxed structures using the Landauer-Buttiker formalism^105–107^ and the semiclassical Boltzmann transport formalism using SMEAGOL^108,109^ and BoltzTraP^88^, respectively, both of which are interfaced with the SIESTA^110^ package. SMEAGOL was used to compute the ballistic transmission, and thereby the ballistic conductance, whereas BoltzTraP was used to compute the Seebeck coefficients and the conductivities at room-temperature under the constant relaxation-time (τ_0_) approximation, for both the systems with and without the stacking fault. Note that under the constant relaxation-time approximation, only the Seebeck coefficient is independent of the relaxation time. Thus, for conductivities (σ) and the corresponding power factor, σ/τ_0_ and S^2^σ/τ_0_ are reported instead.

The pseudopotentials for use within SIESTA were generated using the ATOM program^111^. We used numerical atomic orbitals (NAOs) as the basis set within SIESTA, where an *spd*-basis was used for Tl while only an *sp*-basis was used for Ga and Se. The cutoff-radii were carefully chosen to reproduce the valence band structure of bulk TlGaSe_2_ as benchmarked using the all-electron FHI-AIMS code^112^. All DFT calculations were performed at the PBE-GGA^113^ level of theory.

DFT calculations for computing the energy eigenvalues as inputs to the BoltzTraP code were performed on the relaxed structures using a 45 × 45 × 15 k-mesh. The real-space grid resolution for the electron density was set at a plane-wave energy cutoff of 400 Ry. The self-consistent-field (SCF) convergence was achieved when the maximum difference across all elements of the density matrix was below 1 × 10^−5^. The original k-mesh was interpolated to obtain around 240,000 irreducible k-points using BoltzTraP for computation of all the transport coefficients. All transport coefficients were obtained at 300 K.

DFT calculations combined with the non-equilibrium Green’s function formalism (NEGF) within SMEAGOL were performed using a two-terminal setup with both the left and the right electrodes comprising of bulk TlGaSe_2_ and the central scattering region comprising of the same system with or without the stacking fault. DFT calculations for the idealized semi-infinite leads were performed using a 21 × 21 × 21 k-mesh with a real-space grid resolution set at a plane-wave energy cutoff of 400 Ry and the SCF convergence criterion set at 1 × 10^−4^. The computed self-energies of the leads were then used to compute the equilibrium density matrix and the Green’s function of the full two-terminal device using a 11 × 11 × 1 k-mesh where the transport occurs along the z Cartesian direction. The real-space grid resolution for the electron density was set at a lower plane-wave energy cutoff of 300 Ry while the SCF convergence criterion was kept the same as used in the leads calculations. The equilibrium density matrix was obtained from an integration of the lesser Green’s function along a contour in the complex energy plane, where 32 energy points were used along the semicircle and the imaginary line that form the contour, and we introduced 16 poles.

The electronic band structures and PDOS were calculated via VASP with the same parameters as the stacking fault calculation. The effective mass was obtained from the band structures using the Effective Mass Calculator package^91^. The matrix of effective mass inverse is

$${\left(\frac{1}{{m}^{* }}\right)}_{{ij}}=\frac{1}{{\hslash }^{2}}\frac{{\partial }^{2}E(k)}{\partial {k}_{i}\partial {k}_{j}},i,j=x,y,z$$

where *i* and *j* are reciprocal components and *E*_*n*_*(k)* is the dispersion relation for the *n*-th band. The second order derivatives are then obtained numerically by the finite difference method. The matrix is diagonalized to find the eigenvalues, whose inverse is the primary effective masses.

### Associated content

#### Supporting information

Tables of ground state energy per unit cell and stacking fault energies of TlGaSe_2_, and effective masses along different cartesian directions for *AA*- and *AB*-stacking. Figures for electrical conductance for both with and without stacking faults using SMEAGOL, thermoelectric power factors along the *xx-* and *yy*-directions for *AA*- and *AB*-stacking, thermoelectric power factors along the *xx-* and *yy*-directions vs. hole carrier concentration for *AA*- and *AB*-stacking. Supplementary Dataset 1 includes relaxed *AA*-(bulk), *AB*-, and *AAB*- stacking of TlGaSe_2_ structures as CIF structure files.

## Supplementary information

Supplementary information

Supplemental Dataset 1

## Data Availability

Data is provided within the manuscript or supplementary information files. Any additional data is available from the corresponding authors upon reasonable request.

## Abbreviations

STEM: scanning transmission electron microscopy
DFT: density functional theory
SAED: selected area electron diffraction
