# Supplementary material for: Elucidating the role of stacking faults in TlGaSe2 on its thermoelectric properties
Source: NPJ 2D Mater Appl. 2025 Jun 6;9(1):46. doi: 10.1038/s41699-025-00569-x (PMC12143981; doi:10.1038/s41699-025-00569-x)
Supplement: Supplementary file 1 — Supplementary information [file 41699_2025_569_MOESM1_ESM.pdf]

Supplemental Information for

Elucidating the Role of Stacking Faults in $TlGaSe_2$ on its Thermoelectric Properties

Tigran Simonian[†§], Ahin Roy[†§ ˩], Akash Bajaj[‡§], Rui Dong[‡§], Zheng Lei[Δ], Zdeněk Sofer[Δ], Stefano Sanvito[‡§], Valeria Nicolosi[†§]**

† School of Chemistry, Trinity College Dublin, College Green Dublin 2, D02 PN40, Ireland

‡ School of Physics, Trinity College Dublin, College Green Dublin 2, D02 PN40, Ireland

Δ Department of Inorganic Chemistry, University of Chemistry and Technology Prague, Technická 5, 166 28 Prague 6, Czech Republic

§ Centre for Research on Adaptive Nanostructures and Nanodevices (CRANN), Trinity College Dublin, 43 Pearse St, Dublin 2, D02 W085, Ireland

[˩]Materials Science Centre, Indian Institute of Technology Kharagpur, Kharagpur, West Bengal, IN721302

*Correspondence should be addressed to T.S. (simoniat@tcd.ie) and V.N. (nicolov@tcd.ie)

	AA (bulk)	*AB*	*AAB*
E_0 [eV/unit cell]	-234.75962	-234.57563	-234.57615
γ_{SFE} [mJ m^{-2}]		12.70	12.67

Supplementary Table 1: **Ground state energies per unit cell and stacking fault energies of $TlGaSe_2$.** Table of ground state energy per unit cell (E_0) of bulk (*AA*), *AB*-, and *AAB*-stacking orders of $TlGaSe_2$ calculated using PBE-GGA[1]. The calculated stacking fault energies (γ_{SFE}) for the *AB*- and *AAB*-type structures of $TlGaSe_2$ are also shown.

	AA-Stacking			AB-Stacking		
	xx	*yy*	*zz*	*xx*	*yy*	*zz*
*m** (h)	0.56	0.54	0.11	0.54	0.54	0.10
*m** (e)	0.30	0.23	0.11	0.26	0.22	0.11

Supplementary Table 2: **Effective mass (*m**) of hole (*h*) and electron (*e*) charge carriers in $TlGaSe_2$.** Table of effective masses (*m**) of hole (*h*) and electron (*e*) charge carriers in both bulk (*AA*-) and *AB*-stacking of $TlGaSe_2$, along the major cartesian axes of the unit cell, calculated from their electronic band structures. Values of effective mass are shown in units of mass of free electron (m_0).

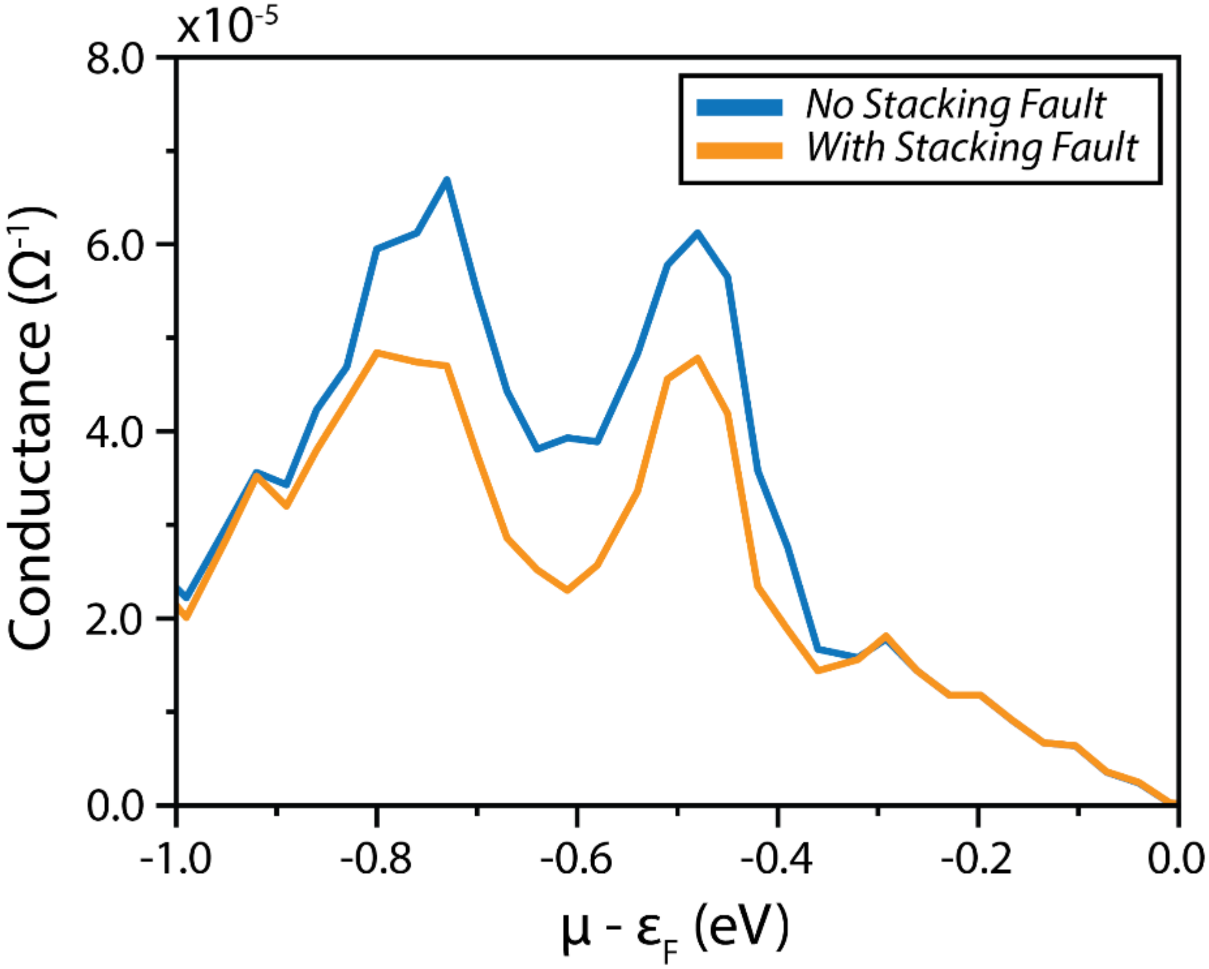

Supplementary Figure S1: **Ballistic conductance calculation of $TlGaSe_2$ along [001] stacking direction**. Ballistic conductance along the [001] stacking direction obtained using SMEAGOL [2,3] for $TlGaSe_2$ with (orange) and without (blue) stacking faults. Only the *p*-doped region (i.e., Fermi level downshifts) is shown here. See Methods in Main Text for calculation details.

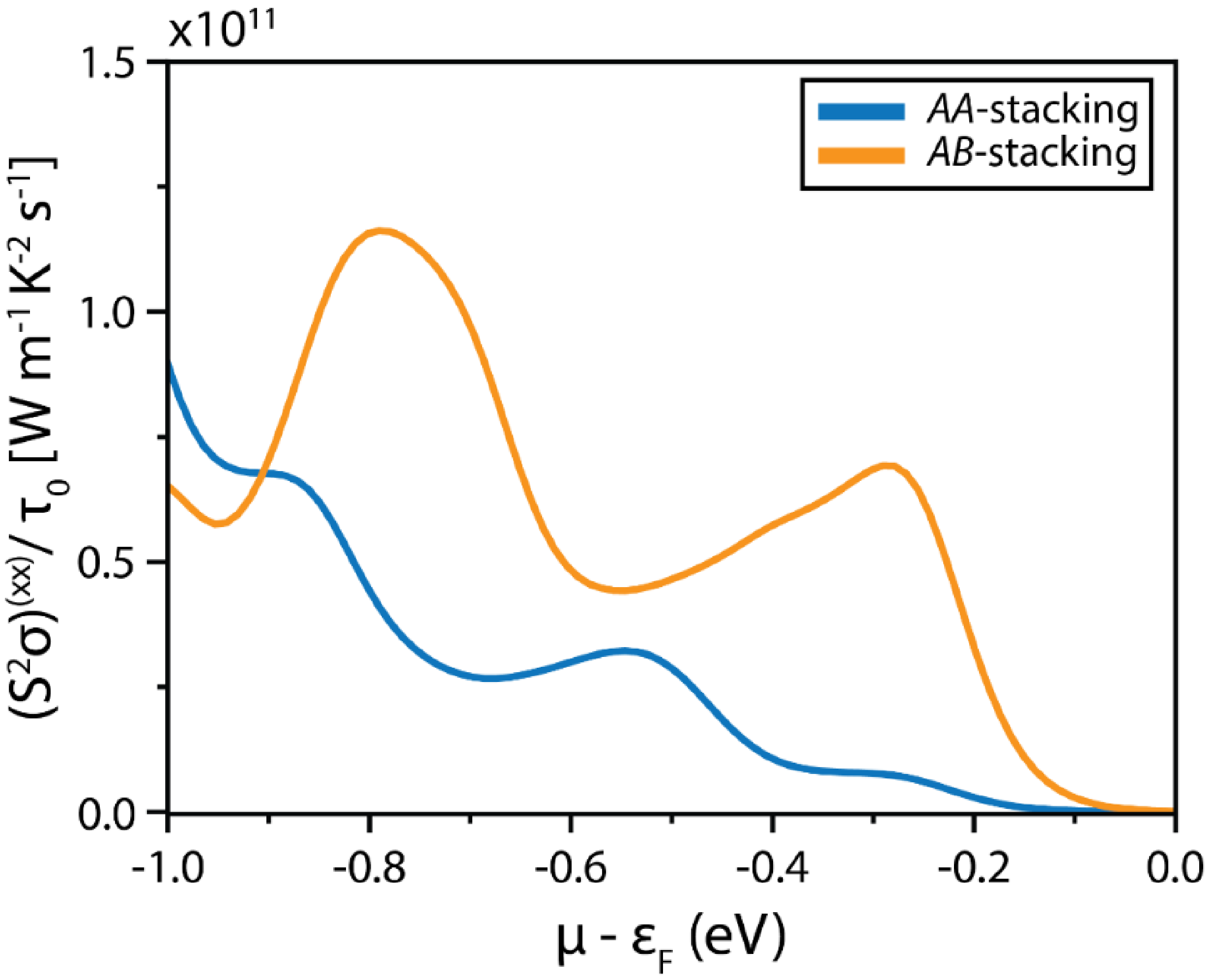

Supplementary Figure S2: **Thermoelectric power factor calculations of $TlGaSe_2$ transverse the [001] stacking direction (*xx*-component).** Thermoelectric power factor transverse to the stacking direction (*xx*-component) for $TlGaSe_2$ for bulk (*AA*-, blue) and *AB*-stacking order (orange), obtained using BoltzTraP [4]. See Methods in Main Text for calculation details.

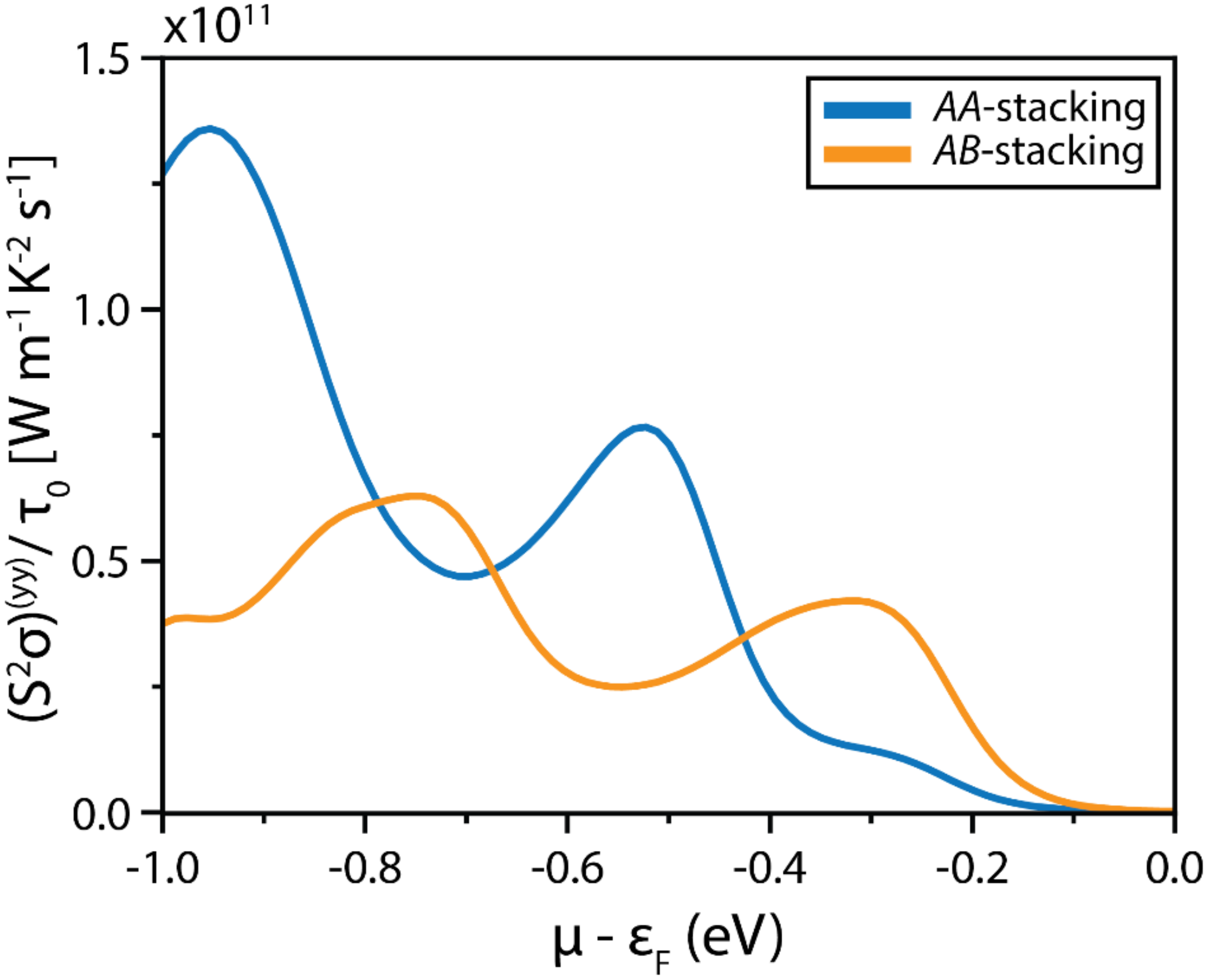

Supplementary Figure S3: **Thermoelectric power factor calculations of $TlGaSe_2$ transverse the [001] stacking direction (*yy*-component).** Thermoelectric power factor transverse to the [001] stacking direction (*yy*-component) for $TlGaSe_2$ for bulk (*AA*-, blue) and *AB*-stacking order (orange), obtained using BoltzTraP [4]. See Methods in Main Text for calculation details.

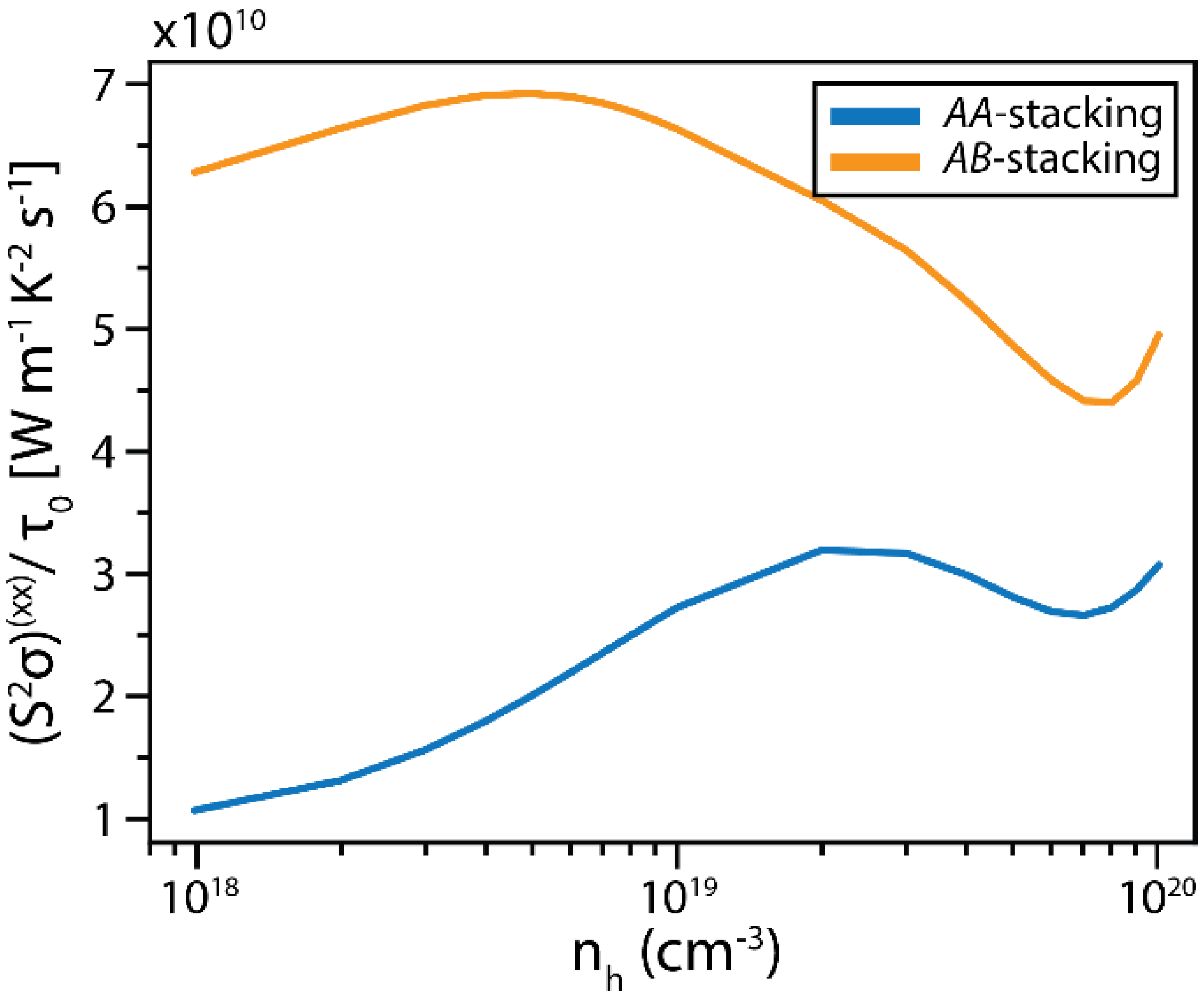

Supplementary Figure S4: **Thermoelectric power factor of $TlGaSe_2$ vs hole carrier concentration (*xx*-component).** Thermoelectric power factor of $TlGaSe_2$ transverse to the [001] stacking direction (*xx*-component) vs hole carrier concentration for bulk (*AA*-, blue) and *AB*-stacking order (orange), obtained using BoltzTraP [4]. See Methods in Main Text for calculation details.

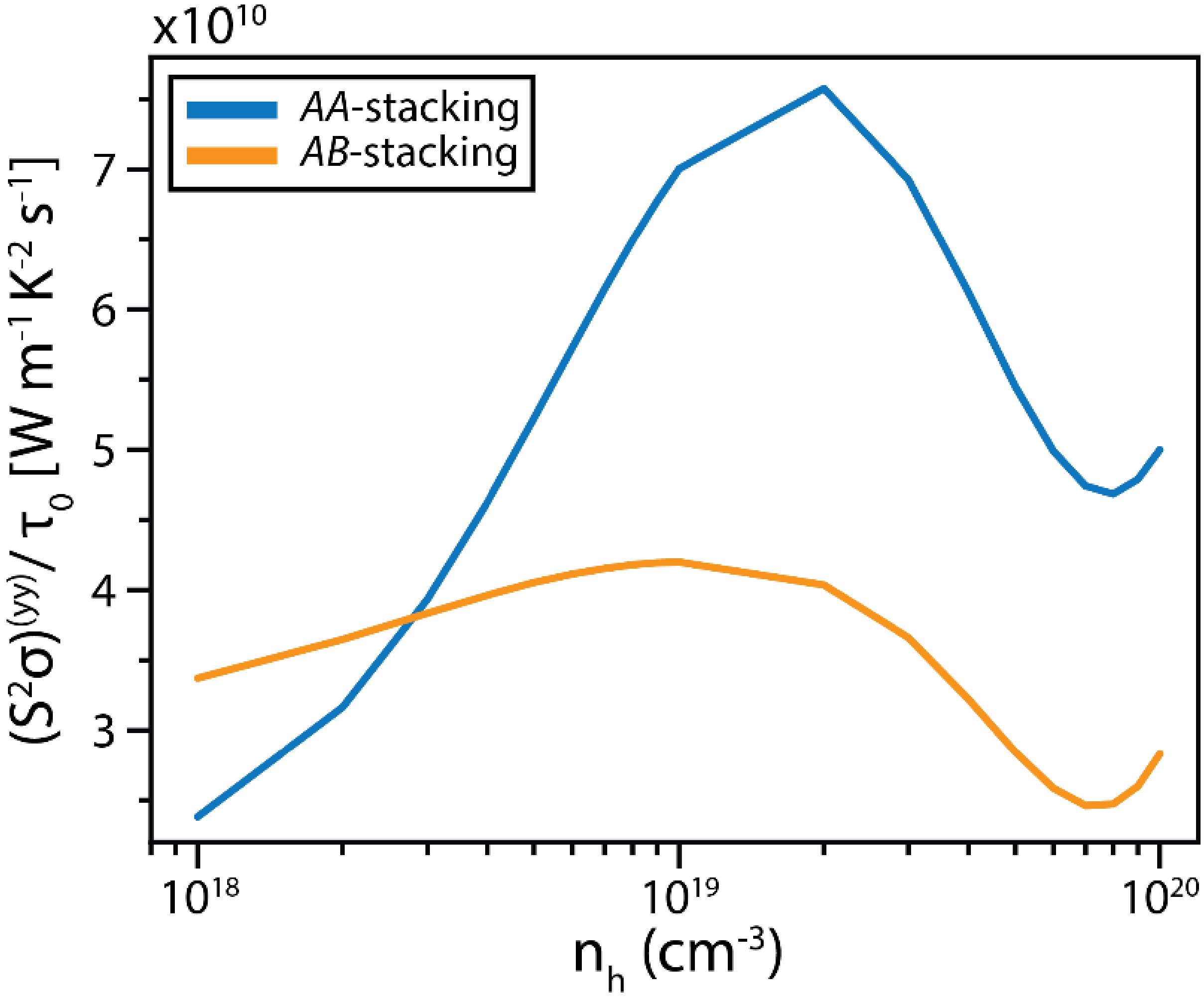

Supplementary Figure S5: **Thermoelectric power factor of $TlGaSe_2$ vs hole carrier concentration (*yy*-component).** Thermoelectric power factor of $TlGaSe_2$ transverse to the [001] stacking direction (*yy*-component) vs hole carrier concentration for bulk (*AA*-, blue) and *AB*-stacking order (orange), obtained using BoltzTraP [4]. See Methods in Main Text for calculation details.

References:

(1) Perdew, J. P.; Burke, K.; Ernzerhof, M. Generalized Gradient Approximation Made Simple. *Phys. Rev. Lett.* **1996**, *77* (18), 3865–3868. https://doi.org/10.1103/PhysRevLett.77.3865.

(2) Rocha, A. R.; García-Suárez, V. M.; Bailey, S.; Lambert, C.; Ferrer, J.; Sanvito, S. Spin and Molecular Electronics in Atomically Generated Orbital Landscapes. *Phys. Rev. B* **2006**, *73* (8), 085414. https://doi.org/10.1103/PhysRevB.73.085414.

(3) Rungger, I.; Sanvito, S. Algorithm for the Construction of Self-Energies for Electronic Transport Calculations Based on Singularity Elimination and Singular Value Decomposition. *Phys. Rev. B* **2008**, *78* (3), 035407. https://doi.org/10.1103/PhysRevB.78.035407.

(4) Madsen, G. K. H.; Carrete, J.; Verstraete, M. J. BoltzTraP2, a Program for Interpolating Band Structures and Calculating Semi-Classical Transport Coefficients. *Comput. Phys. Commun.* **2018**, *231*, 140–145. https://doi.org/10.1016/J.CPC.2018.05.010.
